# A Cross-Scale 3D Attention and Boundary-Aware Network for Sea–Land Segmentation

**DOI:** 10.3390/jimaging12100520

**Published:** 2026-10-19

**Authors:** Yaonan Yan, Xingpeng Yang, Peng Zhao, Weiqing Cheng, Jiating Yang, Xijie Li

**Affiliations:** 1 Air Force Early Warning Academy, Wuhan 430070, China; 947409483@qq.com (Y.Y.); 321500157@qq.com (X.Y.); zip9809@163.com (P.Z.); 27724597@qq.com (W.C.); 2 State Key Laboratory of Ultrafast Optical Science and Technology, Xi’an Institute of Optics and Precision Mechanics, Chinese Academy of Sciences, Xi’an 710119, China; yangjiating@opt.ac.cn; 3 Shaanxi Key Laboratory of Optical Remote Sensing and Intelligent Information Processing, Xi’an Institute of Optics and Precision Mechanics, Chinese Academy of Sciences, Xi’an 710119, China

**Keywords:** deep learning (DL), sea–land segmentation, 3D attention fusion, boundary awareness

## Abstract

Coastal segmentation struggles with complex terrain configurations and discontinuous edge demarcation. To resolve these challenges, we propose a novel deep learning model, the Cross-Scale Attention and Boundary-Aware Network (CABANet). The network synergistically enhances segmentation performance through two core innovations. First, we design a Cross-Scale 3D Attention Fusion Module, which creatively stacks multi-scale 2D feature maps from the backbone into a pseudo-3D feature volume. It then performs efficient contextual modeling across different geographic scales via a decomposed 3D attention mechanism, generating a highly optimized semantic summary feature. Second, we design a boundary enhancement module to strengthen the extraction and analysis of boundary features and improve the segmentation of the sea–land boundary, while utilizing contextual information to guide the boundary enhancement process. Evaluations on Landsat-8 OLI and SLSD datasets confirm state-of-the-art performance in both regional homogeneity and edge continuity.

## 1. Introduction

The deepening exploration of marine resources has propelled widespread attention toward remote sensing-based semantic segmentation of sea–land scenes in marine science and engineering. This technology plays an indispensable role in marine resource surveys, environmental monitoring, and disaster early warning systems. It not only provides foundational data support for the rational exploitation of marine resources but also offers reliable technical means for marine environmental protection and management. However, the complex characteristics of sea–land segmentation images such as illumination variations and dynamic wave patterns often lead to low accuracy and poor noise resistance in traditional image feature-based segmentation methods, making them inadequate for practical requirements [1].

To address the limitations of conventional approaches, researchers have developed numerous improved algorithms based on remote sensing data, which can be broadly categorized into two groups: traditional image processing methods and deep learning-based methods. Traditional methods primarily rely on image features like color, texture, and shape, with representative techniques spanning multiple approaches. Firstly, threshold segmentation techniques have been widely adopted: for instance, You et al. [2] proposed an adaptive statistical model-based thresholding scheme that corrects misclassified land regions by analyzing variance differences between sea and land statistical models, while Otsu [3] introduced an adaptive thresholding method applicable to foreground–background separation in sea–land segmentation. Building on feature-based analysis, Liu et al. [4] developed a dense point matching-based scene alignment method to extract discriminative texture features for segmentation tasks. Expanding into morphological processing, Suzuki et al. [5] devised a boundary-tracking topological analysis method to enhance morphological boundary extraction. Finally, to address region coherence challenges, Zha et al. [6] designed a spatial constraint model-based superpixel region merging method, specifically tailored for sea–land segmentation in maritime target detection scenarios.

In the realm of deep learning, researchers have constructed more efficient architectures for general remote sensing scene interpretation. Chen et al. [7] designed MSDCNet, a multilevel spatial distribution-enhanced coupled network for ship counting in remote sensing images; Zhu et al. [8] introduced CDSANet, demonstrating that combining convolutional, Transformer, and attention branches can effectively improve the accuracy of ship instance segmentation, and Lu et al. [9] introduced a multibranch fusion-based feature enhancement network for remote-sensing scene classification. In addition, Moussaid et al. [10] proposed an unsupervised learning framework to segment and classify tree crowns in overlapping orchards from satellite imagery; Pan et al. [11] proposed a generic and automatic approach using fully convolutional neural networks to extract road networks from very high-resolution remote sensing images; Cao et al. [12] designed DFENet, a novel dual-path feature extraction network that learns complementary global and local representations for semantic segmentation of remote sensing images. Furthermore, Huynh-The et al. [13] introduced a novel semantic segmentation architecture, HBSeNet, which combines context and spatial paths to optimize accuracy and complexity in remote sensing image segmentation, achieving superior performance compared to state-of-the-art models. These innovative methods leverage the powerful feature learning capabilities of deep neural networks to effectively overcome the poor environmental adaptability of traditional approaches, laying a solid methodological foundation for sea–land segmentation.

Motivated by these advances, deep learning has been increasingly introduced to sea–land segmentation. Among mainstream approaches, convolutional neural network (CNN)-based architectures such as U-Net [14] and Fully Convolutional Networks (FCN) [15] excel in learning complex features through end-to-end training, achieving robust segmentation performance. On this basis, dedicated sea–land and coastline segmentation networks have been developed: Cui et al. [16] created a scale-adaptive network tailored for remote sensing sea–land segmentation; Aghdami et al. [17] enhanced Shoreline Segmentation by adapting Standard U-Net for precise coastline mapping; Chu et al. [18] proposed Res-UNet, which integrates ResNet with conditional random fields and morphological operations to refine coastline delineation; and Cheng et al. [19] introduced a deep CNN incorporating local smoothing regularization and multi-task loss to improve boundary quality. Beyond purely optical CNN pipelines, other studies have targeted the harsh imaging conditions of maritime scenes: Wang et al. [20] proposed MCAT-UNet, an efficient semantic segmentation architecture for high-resolution remote sensing images, combining multi-scale convolutional attention and cross-shaped window transformers to improve feature learning, boundary distinction, and computational efficiency; Xu et al. [21] developed a clutter differentiation technique using multiframe echo characteristics, and Wang et al. [22] proposed the Multi-Weather DomainShifter, an LLM-based transfer agent that alleviates the domain shift caused by complex weather in aerial image processing. Despite this progress, their performance in complex coastal scenes remains limited, as discussed below.

**Figure 1:**
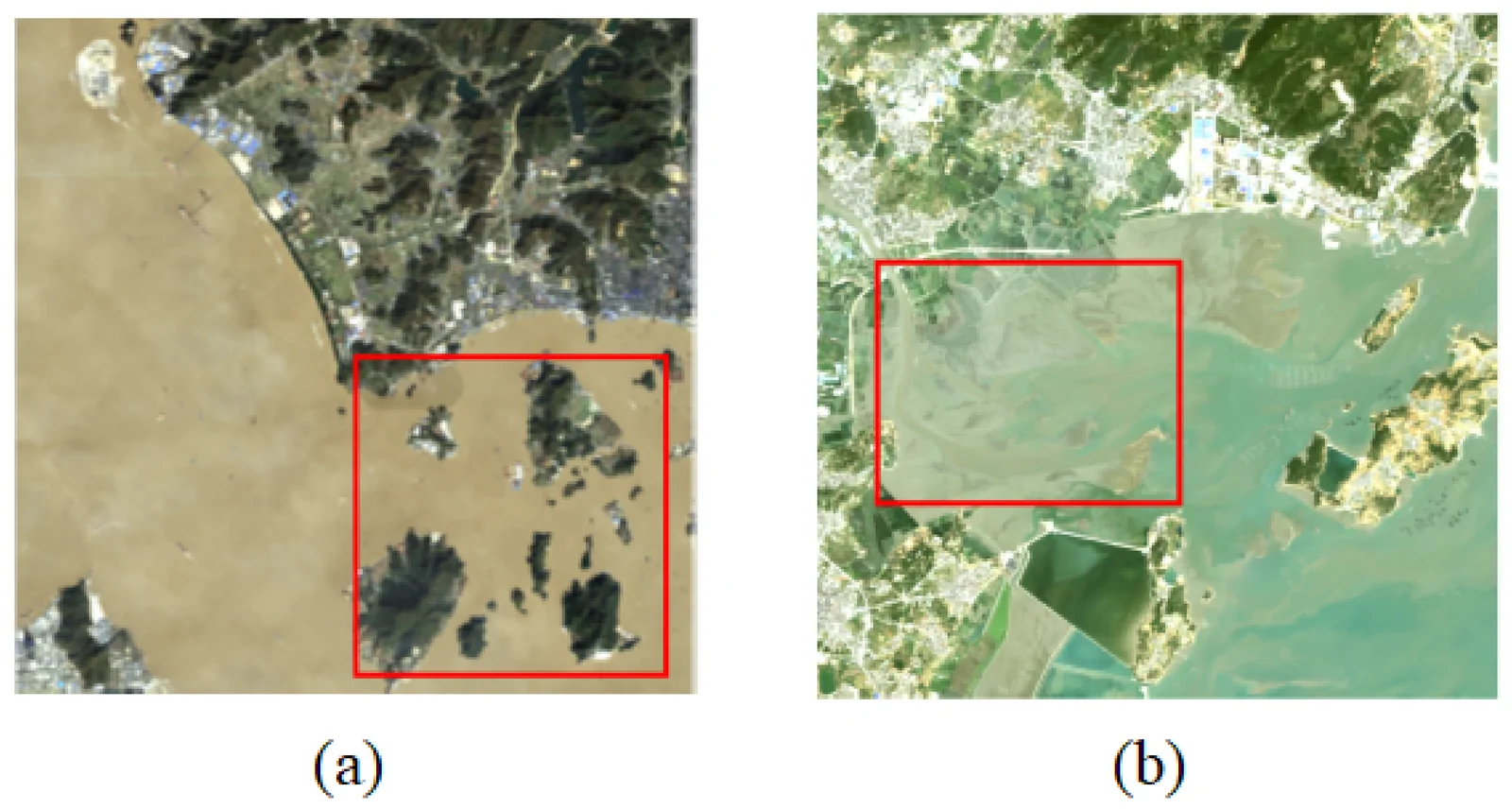
The coastal area from the Landsat-8 OLI dataset. (**a**) shows the complex features of the rocky coast, including small islands and irregular reefs. (**b**) shows the characteristics of the mudflat coast. The contrast of the sea–land boundary is low and presents a continuous weak boundary. The red boxes highlight the typical coastal regions in (**a**,**b**).

Remote sensing imagery of coastal zones presents significant challenges due to the complex geomorphological features along sea–land boundaries. As illustrated in Figure 1a, rocky coastal areas exhibit numerous small, irregularly shaped offshore islands and reefs that create intricate spatial patterns [16,18]. These features often demonstrate ambiguous texture characteristics in satellite imagery, leading to frequent segmentation failures in existing deep learning models [17,19]. While architectures like U-Net have advanced segmentation accuracy, they still struggle with boundary discontinuities and noise susceptibility in dynamic coastal environments, which has motivated a series of boundary-aware segmentation studies [23,24,25]. Indeed, this challenge remains open: even the most recent efforts, such as the prompt mask-attention framework of Ji et al. [26] and the lightweight dual-branch TCUNet of Xiong et al. [27] were specifically designed to better capture weak and irregular sea–land transitions in complex coastal scenes.

The complexity further intensifies in mudflat coastal regions (Figure 1b), where gently sloping terrains exhibit low optical contrast between sea and land [17,19]. The unique physical properties of these areas result in weakly defined boundaries with gradual transitions, making traditional segmentation methods [3,5] prone to erroneous delineation. Such challenges underscore the need for specialized solutions in coastal remote sensing analysis.

To address these limitations, we introduce a framework with three key innovations:

1. We propose a Cross-Scale 3D Attention Fusion Module aimed at enhancing the attention to regional features of sea and land in remote sensing images, while suppressing irrelevant features in the sea and land images.
2. In order to enhance the extraction and analysis of boundary features and improve the segmentation accuracy of the model at the boundary of sea and land regions, we propose a boundary awareness enhancement module.
3. Since the binary cross-entropy loss function cannot solve the problem of class imbalance in boundary segmentation, a weighted binary cross-entropy loss function $L_{WBCE}$ is introduced into the boundary loss function proposed in this algorithm. At the same time, in order to effectively constrain the boundary region, we add the boundary-F1 loss function to the boundary loss function to improve and optimize the quality of boundary segmentation.

The structure of this paper is organized as follows: Section 2 discusses the related works about the methods of the boundary enhancement and attention mechanism. Section 3 discusses the proposed algorithm for sea–land segmentation. Section 4 presents the experimental results of the proposed method on two publicly available land and sea segmentation datasets. And Section 5 summarizes the proposed method.

## 2. Related Work

In this section, we briefly review the relevant work on attention mechanisms and boundary-enhanced mechanisms. Attention mechanisms and boundary awareness enhancement are important technologies in the task of land and sea segmentation in remote sensing images. By integrating context features at different scales and stages through an attention mechanism, boundary awareness enhancement provides edge information to improve the segmentation accuracy of land and sea boundaries.

### 2.1. Attention Mechanism

In sea–land segmentation tasks, attention mechanisms significantly improve segmentation performance through core functionalities such as enhancing feature representation, suppressing background interference, promoting multi-scale feature fusion, increasing model robustness, and integrating cross-channel information. Specifically, these mechanisms strengthen semantic expression in critical regions through dynamic allocation of feature weights. For instance, Wu et al. [28] proposed CMLFormer, a novel CNN and multi-scale local-context transformer network for remote sensing image semantic segmentation, which effectively captures both local and global feature information with low complexity, demonstrating excellent performance on the Vaihingen and Potsdam datasets. Zhang et al. [29] designed a global–local adaptive attention mechanism that explores semantic dependencies between different classes while preserving their distinctiveness. To better capture global contextual information, Chen et al. [30] integrated a hybrid attention mechanism with Transformer, utilizing channel-spatial transformation modules and adaptive weighting strategies to enhance feature representation. Huang et al. [31] introduced a cross-attention module in CCNet, enabling efficient full-image dependency modeling for contextual information. For multi-scale semantic fusion, Zeng et al. [32] developed an MSI module incorporating efficient cross-attention to replace traditional skip connections, Ding et al. [33] proposed a semantic attention enhancement (SAB) framework that reinforces semantic consistency through adaptive fusion of cross-layer contextual information, while Zhang et al. [34] proposed SSPNet, a spatial–spectral perception network that couples position encoding with attention mechanisms to enhance spatial–spectral feature extraction. Zhang et al. [35] designed a cascaded spatial cross-attention network that progressively strengthens spatial–spectral representation learning. Moreover, attention-based architectures have been extended to cross-modal remote sensing tasks: Zhao et al. [36] developed a context-driven sparse decoding framework for remote sensing visual grounding, and Huang et al. [37] proposed ReCoTR, which reduces the semantic cognitive shift via dual-consensus token compression for remote sensing image–text retrieval. Also, Yang et al. [38] introduced the Correlated Mapping Attention Cooperative Network (CMACNet), a novel framework that addresses the challenge of segmenting spectrally similar objects in remote sensing by extending the segmentation from the image domain to the feature domain using an autoencoder and correlated mapping attention mechanism.

Notably, attention mechanisms often entail high computational complexity and data requirements. To address this, researchers focus on developing lightweight solutions to balance performance and efficiency. For example, Shi et al. [39] proposed a lightweight multi-scale attention decoder that achieves efficient multi-scale feature processing and spatial detail recovery with reduced parameter counts, offering practical deployment advantages. These innovations demonstrate that through structural optimization and functional expansion, attention mechanisms can enhance segmentation accuracy while meeting computational constraints for real-world applications.

### 2.2. Boundary Enhancement

Boundary enhancement techniques play a pivotal role in improving segmentation precision by explicitly modeling geometric discontinuities across diverse targets, including buildings, water bodies, and vegetation. In complex geographical contexts, such as meandering river networks, irregular coastlines, and dense urban layouts, these methods effectively mitigate boundary ambiguity and topological errors. Traditional approaches like the Canny edge detector [40] laid foundational principles for gradient-based edge extraction, remaining relevant in tasks like coastal boundary delineation. Building upon these fundamentals, modern learning-based frameworks introduce sophisticated enhancements: Jung et al. [41] developed a recursive refinement mechanism specifically for urban building edges in remote sensing imagery, while Ding et al. [42] proposed ASLNet, which synergizes adversarial shape learning with CNN regularization to resolve boundary blurring. For weakly supervised scenarios, Rong et al. [43] established a cooperative training paradigm where boundary constraints and semantic predictions mutually reinforce segmentation accuracy. Beyond structural targets, Xu et al. [44] designed a multi-scale edge hierarchy to precisely demarcate agricultural parcels.

In the task of sea–land segmentation, enhancing boundary clarity between land and sea is crucial for improving segmentation accuracy, which is particularly important for applications such as sea target detection and coastline extraction. Several advanced methods have been proposed to address the challenges in this domain. Wang et al. [45] introduced a boundary-guided multilevel feature fusion module and a spatial semantic context aggregation module based on channel correlation, effectively tackling the issues of feature fusion and context aggregation in remote sensing image segmentation. Similarly, Xu et al. [23] developed HBCNet, a network that integrates boundary information with semantic features, achieving state-of-the-art performance in remote sensing image segmentation. Additionally, Chen et al. [46] proposed the multilevel context-attention segmentation network (MCSNet), which combines boundary detection and semantic segmentation, ensuring a comprehensive and robust segmentation capability. Zhao et al. [24] also proposed a novel multi-scale feature weighted-aggregating and boundary enhancement network (MFBE-Net) to improve the segmentation of high-resolution remote sensing images (HRRSIs) by effectively addressing issues with object size variation, boundary clarity, and information redundancy. These advancements collectively contribute to more accurate and reliable sea–land segmentation.

## 3. Method

To solve the problems existing in Figure 1, we propose a sea–land segmentation algorithm based on Cross-Scale 3D Attention Fusion Module and Boundary Awareness Enhancement. This section introduces the sea–land segmentation algorithm proposed in this paper in detail.

**Figure 2:**
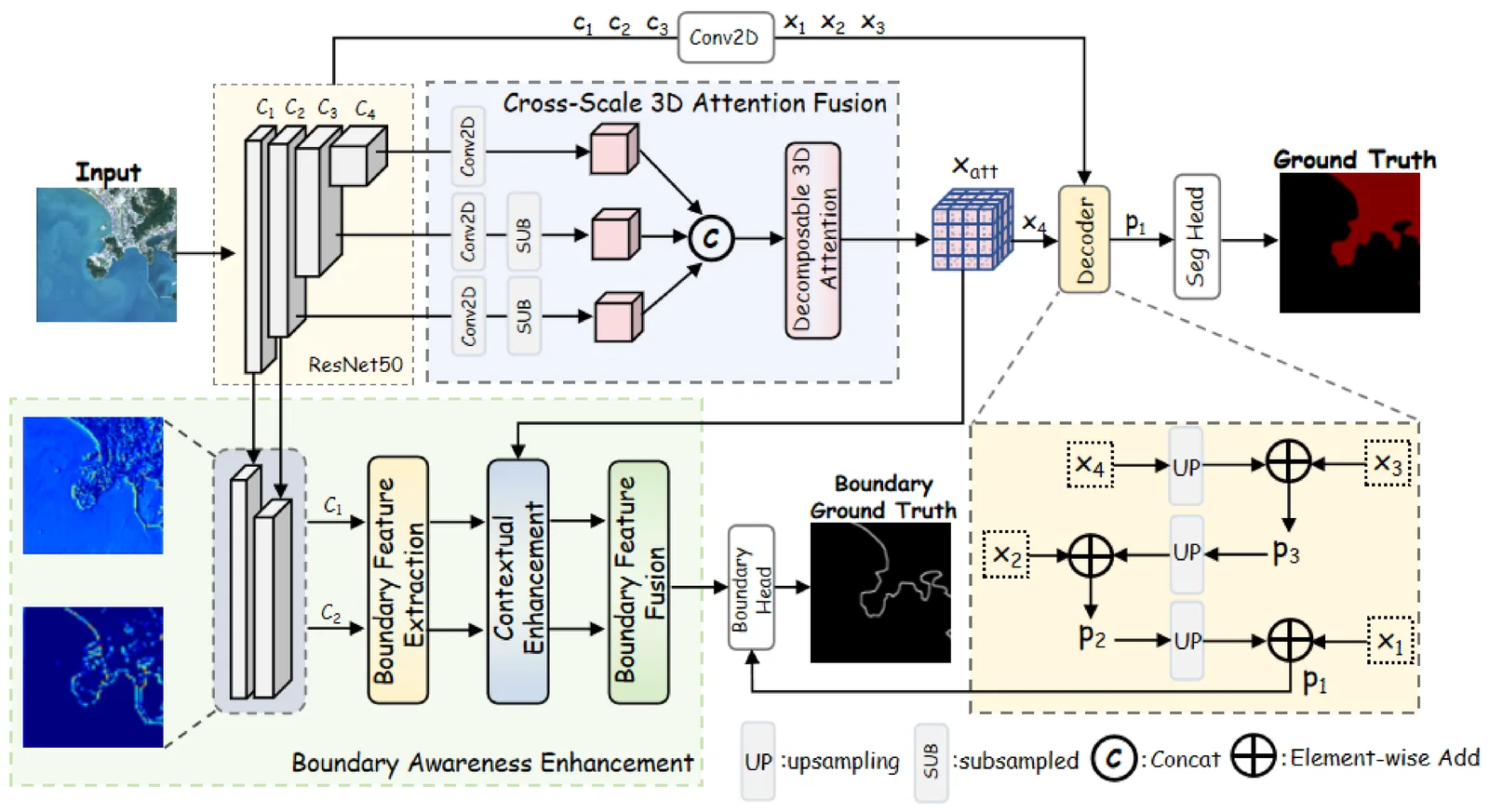
The network structure of the sea–land segmentation algorithm. The overall network structure is mainly composed of four parts: the backbone feature encoder, the Cross-Scale 3D Attention Fusion Module for deep fusion of multi-scale context, the boundary-aware decoder specifically designed for coastline localization, guided by high-level context, and the cascaded decoding and multi-task supervision framework.

Figure 2 shows the network structure diagram of the sea–land segmentation algorithm. The overall network structure is designed based on the encoder–decoder architecture, which is mainly composed of four parts: the backbone feature encoder, the Cross-Scale 3D Attention Fusion Module for deep fusion of multi-scale context, the boundary-aware decoder specifically designed for coastline localization, guided by high-level context, and the cascaded decoding and multi-task supervision framework.

In the encoding stage, the sea–land images with resolution H×W are input into the pre-trained ResNet50 [47] backbone. The residual blocks in ResNet50 are used to extract the features of four different stages, denoted as $F_{i}\in R^{\frac{H}{2^{i+1}}\times \frac{W}{2^{i+1}}\times C_{i}},\;i=1,2,3,4.$ where $C_{i}$ indicates the number of channels at stage *i*. By using ResNet50 to extract different hierarchical features of images layer by layer, from low-level texture information to high-level semantic information, fine-grained boundary and regional changes can be captured in the sea–land segmentation task. Its deep network structure facilitates the identification of complex ground features, especially in the sea–land boundary transition and segmentation of different ground types.

In the stage of decoding, the extracted deep features are input into the Cross-Scale 3D Attention Module to strengthen the network’s ability to capture the context information of sea and land images. Through the Cross-Scale 3D Attention Module, the irrelevant features of sea–land coastal zones and offshore islands and reefs can be effectively suppressed, and the attention to the features of sea–land boundary regions can be increased, so that the network can more accurately identify and process sea–land images and improve the overall segmentation accuracy. Then, the features in the decoder are upsampled stage-by-stage to recover the resolution of the feature map. After the feature map is upsampled to the size of the original image, the decoded features and the enhanced features of boundary awareness are further fused and input into the segmentation head together to predict the segmentation results, then calculate the segmentation loss.

The proposed method enhances sea–land segmentation by leveraging shallow features ($C_{1}$ and $C_{2}$) from ResNet50’s first and second stages, which capture detailed information like color, texture, and boundaries. These shallow features are processed by a Boundary Awareness Enhancement Module, guided by deep features with Cross-Scale 3D Attention to improve boundary perception. The enhanced features are fused with decoder outputs, improving boundary awareness during segmentation. Additionally, a boundary head is added in the decoding stage to predict boundary segmentation. The network jointly optimizes both segmentation and boundary loss functions, promoting information complementarity between sea–land segmentation and boundary segmentation, thus improving the model’s generalization ability.

### 3.1. Cross-Scale 3D Attention Fusion Module

**Figure 3:**
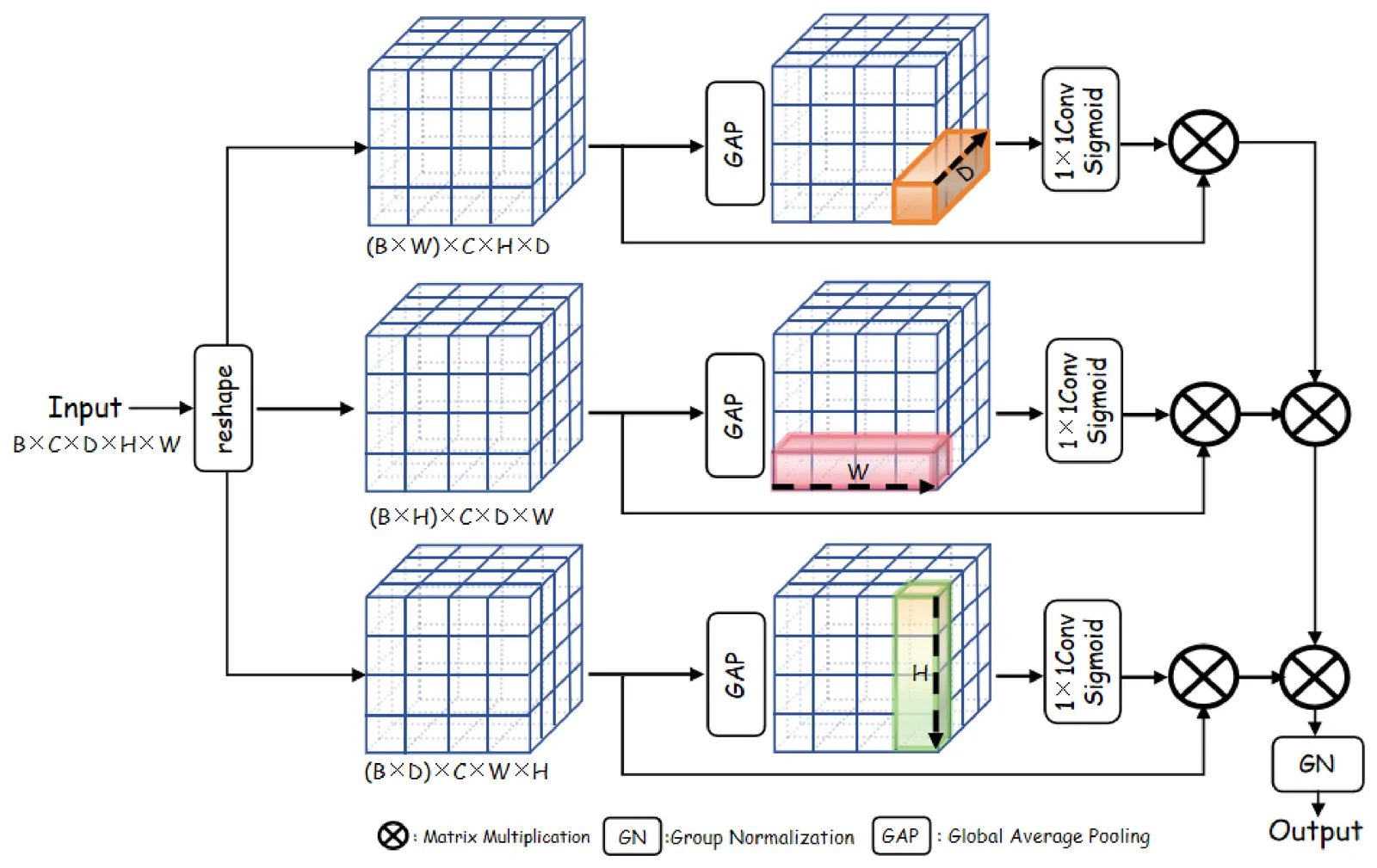
Cross-Scale 3D Attention Fusion Module. This module stacks multi-scale 2D feature maps into pseudo-3D feature volumes and efficiently models Cross-Scale and spatial contextual relationships by computing 3D attention through decomposition across three orthogonal planes; the dashed arrows denote the D, W, and H dimensions covered by the three attention planes.

To effectively fuse the multi-scale features extracted by the backbone and overcome the insufficient contextual interaction in traditional fusion methods (e.g., feature concatenation or addition), we propose a novel Cross-Scale 3D Attention Fusion Module (as shown in Figure 3). The core idea of this module is to stack the multi-scale 2D feature maps $\left\{F_{2},F_{3},F_{4}\right\}$ into a pseudo-3D feature volume and perform contextual modeling in this higher-dimensional space. Unlike conventional multi-scale fusion strategies that aggregate features through 2D concatenation, element-wise addition, or skip connections, our module treats the scale dimension as a first-class axis of the representation. The decomposed D-H and D-W attention planes explicitly model cross-scale dependencies at each spatial location, so that the importance of each geographic scale is adaptively re-weighted according to the spatial content, while the H-W plane simultaneously refines the spatial context within each scale. Moreover, since the attention is decomposed into three orthogonal 2D planes, the computational cost grows linearly with the volume size rather than cubically as in full 3D attention.

First, we unify the channel dimensions of the feature maps $\left\{F_{2},F_{3},F_{4}\right\}$ to $C^{\prime }$ = 512 using parallel 1×1 convolutions. Subsequently, for spatial alignment, we downsample the higher-resolution features $F_{2}^{\prime }$ and $F_{3}^{\prime }$ to the same size as $F_{4}^{\prime }$, which is $\left(\frac{H}{32},\frac{W}{32}\right)$, via bilinear interpolation. The aligned feature maps are denoted as $\left\{{\hat{F}}_{2}^{\prime },{\hat{F}}_{3}^{\prime },F_{4}^{\prime }\right\}$. Finally, we stack these three feature maps along a new “depth” dimension to construct a pseudo-3D feature volume:

(1) $$V\in R^{C^{\prime }\times 3\times \frac{H}{32}\times \frac{W}{32}},$$

where the depth dimension D=3 represents three distinct geographic observation scales. Directly applying attention in a 3D space is computationally expensive. Therefore, we adopt a decomposition strategy that breaks down the 3D attention computation into three orthogonal 2D planes: Depth-Height (D-H), Depth-Width (D-W), and Height-Width (H-W). For the D-H plane, for instance, we first reshape the 3D volume into a 4D tensor view suitable for 2D convolutions by permuting its dimensions. Then, an attention weight map $A_{dh}$ for this plane is generated through average pooling, a 1×1 convolution, and a Sigmoid activation function:

(2) $$A_{dh}=\sigma \left({\mathrm{Conv}}_{1\times 1}\left({\mathrm{Pool}}_{\mathrm{avg}}\left(V_{dh}\right)\right)\right).$$

The re-weighted features are obtained by element-wise multiplication with the original features. After performing this operation on all three planes, we fuse their outputs via element-wise addition and Group Normalization:

(3) $$V_{\mathrm{out}}=\mathrm{GN}\left(V_{dh}^{\prime }+V_{dw}^{\prime }+V_{hw}^{\prime }\right).$$

Finally, we average-pool $V_{\mathrm{out}}$ along the depth dimension to generate a 2D geographic contextual summary feature map:

(4) $$F_{\mathrm{attn}}\in R^{\frac{H}{32}\times \frac{W}{32}\times C^{\prime }}.$$

This feature map dynamically integrates multi-scale sea–land region features and serves as a key input for the subsequent Contextual Enhancement Unit.

### 3.2. Boundary Awareness Enhancement

In high-resolution remote sensing images, the sea–land boundary can become blurred due to factors like light, shadow, clouds, and fog, resulting in inaccurate segmentation. Compared to regular images, the boundaries between land objects in remote sensing images are often unclear, making boundary features essential for distinguishing different regions and improving segmentation accuracy. To enhance the extraction and analysis of boundary features and improve sea–land boundary segmentation, we propose a Boundary Awareness Enhancement Module.

**Figure 4:**
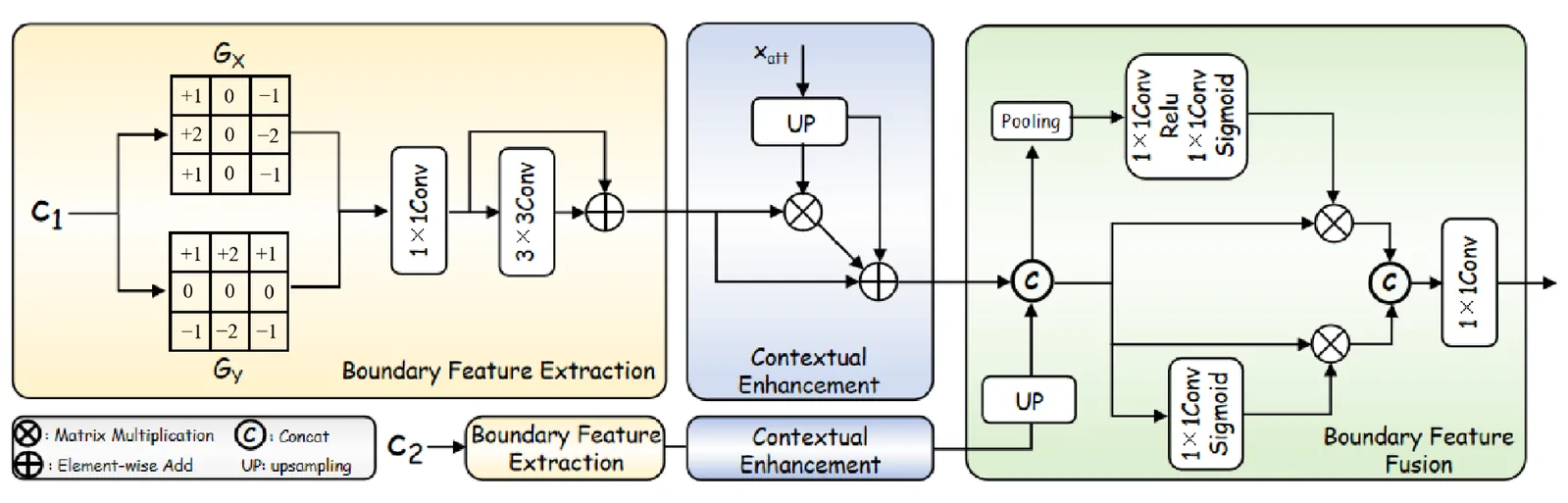
Boundary Awareness Enhancement Module. The Boundary Awareness Enhancement Module consists of three units, Boundary Awareness Unit, Contextual Enhancement Unit, and Boundary Feature Fusion Unit, which are used to extract, enhance, and fuse boundary features to guide the sea–land segmentation and improve the segmentation effect of boundary.

As shown in Figure 4, the Boundary Awareness Enhancement Module comprises three units: (1) Boundary Awareness Unit, (2) Contextual Enhancement Unit, and (3) Boundary Feature Fusion Unit. Initially, shallow features $F_{1}$ and $F_{2}$ from the backbone are extracted. These are then enhanced using the deep semantic feature $X_{attn}$. Finally, the enhanced boundary features are fused and fed into both the segmentation decoder and the boundary head. The Boundary Feature Fusion Unit helps guide and refine the segmentation results, significantly improving the accuracy of sea–land boundary segmentation, especially in coastal regions.

1. Boundary Awareness Unit

In the Boundary Awareness Enhancement Module, in order to be able to effectively extract the boundary features in the shallow features and perceptually enhance the boundary features, we propose the Boundary Awareness Unit, as shown in the yellow module in Figure 4.

As a traditional edge detection method, the Sobel operator identifies the edge by calculating the approximate gradient of the change of the image brightness value, and can well extract the horizontal and vertical edges in the image. In the Boundary Awareness Unit, the Sobel operator and two 3×3 convolutional layers are introduced to realize the identification and awareness of the boundary by the network. The network identifies the boundary information in the feature map through the Sobel operator, while the two 3×3 convolutions can learn and extract more complex boundary features in the image, which helps to retain the key boundary information in the feature map. By stacking multiple convolutional layers, the network is able to gradually build up from simple to complex boundary feature representations.

Specifically, the shallow features $F_{in}\in R^{C_{in}\times H\times W}$ are obtained from the backbone, and Sobel convolution is performed on the shallow features in the horizontal direction and vertical direction respectively to obtain the horizontal gradient value $G_{x}$ and the vertical gradient value $G_{y}$. The gradients of the two directions are integrated by L2 norm to obtain the shallow feature gradient *G*. The specific definition and calculation process are as Equations (5) and (6):

(5) $$\begin{array}{c} G_{x}=\left[\begin{array}{ccc} 1 & 0 & -1\\ 2 & 0 & -2\\ 1 & 0 & -1 \end{array}\right],G_{y}=\left[\begin{array}{ccc} 1 & 2 & 1\\ 0 & 0 & 0\\ -1 & -2 & -1 \end{array}\right] \end{array}$$

(6) $$\begin{array}{c} G=\sqrt{G_{x}^{2}+G_{y}^{2}} \end{array}$$

where $G_{x}$ and $G_{y}$ represent the fixed parameters of the horizontal and vertical Sobel convolution, respectively.

The gradient value after the activation function and the shallow feature $F_{in}$ are multiplied element-by-element, and finally the boundary basic feature $F_{base}\in R^{C_{in}\times H\times W}$ is obtained. The calculation is as Equation (7):

(7) $$\begin{array}{c} F_{\mathrm{base}}=F_{\mathrm{in}}⊙\sigma \left(G\right) \end{array}$$

where ⊙ represents element-wise multiplication, and σ(·) represents the Sigmoid activation function.

After extracting the boundary basic features of the shallow features by Sobel operator, the shallow boundary basic feature $F_{base}$ is used to reduce the channel dimension by 1×1 convolution, and then input into two 3×3 convolutions, and the original boundary basic features are connected by residual connections. Through the stacked convolution blocks, the representation of feature boundary information is strengthened, and the boundary-aware feature $F_{a}\in R^{C\times H\times W}$ is obtained. The calculation is as shown in Equations (8) and (9):

(8) $$\begin{array}{c} {\hat{F}}_{\mathrm{base}}={\mathrm{Conv}}_{1\times 1}\left(F_{\mathrm{base}}\right) \end{array}$$

(9) $$\begin{array}{c} F_{a}={\mathrm{Conv}}_{3\times 3}\left({\mathrm{Conv}}_{3\times 3}\left({\hat{F}}_{\mathrm{base}}\right)\right)+{\hat{F}}_{\mathrm{base}} \end{array}$$

where ${\hat{F}}_{\mathrm{base}}$ is the boundary basis feature after dimension reduction.

In summary, the Boundary Awareness Unit effectively combines the Sobel operator and convolution, and the network can extract image boundary and texture features complementary to each other. The module enhances the representation and recognition ability of the model for boundary features by continuously refining the boundary features, so as to improve the accuracy and robustness of boundary extraction, which is particularly important for sea–land segmentation tasks that require accurate boundary location.

2. Contextual Enhancement Unit

As shown in Figure 4, the method introduces a Contextual Enhancement Unit into the Boundary Awareness Enhancement Module. This module uses deep features enhanced by Cross-Scale 3D Attention to guide and semantically enhance boundary awareness features. Specifically, the boundary aware feature $F_{a}$ is used as the input of the Contextual Enhancement Unit, the deep semantic feature $X_{attn}$ enhanced by Cross-Scale 3D Attention is upsampled, and the feature element-by-element multiplication is performed with the boundary aware feature $F_{a}$, and then the residual connection is used to retain the deep features and shallow features at the same time. The semantically enhanced boundary feature $F_{s}\in R^{C\times H\times W}$ is obtained. The specific calculation process is as shown in Equation (10):

(10) $$\begin{array}{c} F_{s}=F_{a}⊙Up\left(X_{attn}\right)+F_{a}+Up\left(X_{attn}\right) \end{array}$$

where $Up(X_{attn})$ represents an upsampling operation.

3. Boundary Feature Fusion Unit

After generating refined boundary-aware features at different scales through the Boundary Awareness Unit and the Contextual Enhancement Unit, it is crucial to effectively fuse these multilevel features to produce a unified and robust coastline prediction. To achieve this, we designed the Boundary Feature Fusion Unit. As illustrated in the green module in Figure 4, this unit is responsible for integrating the semantically enhanced boundary features from the low-level stages ($F_{1}$ and $F_{2}$) and further refining the fused representation before final prediction.

The low-level features $F_{1}$ and $F_{2}$ capture complementary boundary information. $F_{1}$, with its higher spatial resolution ($\frac{H}{4}\times \frac{W}{4}$), preserves fine-grained details and intricate coastline contours. In contrast, $F_{2}$, with a larger receptive field, provides more contextualized edge information. Fusing them allows the model to leverage the strengths of both scales.

Specifically, we take the two semantically enhanced boundary features, $F_{s,1}\in R^{C\times \frac{H}{4}\times \frac{W}{4}}$ (derived from $F_{1}$) and $F_{s,2}\in R^{C\times \frac{H}{8}\times \frac{W}{8}}$ (derived from $F_{2}$), as inputs. To enable fusion, we first upsample the lower-resolution feature $F_{s,2}$ to match the spatial dimensions of $F_{s,1}$ using bilinear interpolation. Then, we concatenate these two features along the channel dimension, creating a rich, multi-scale boundary feature representation $F_{\mathrm{concat}}\in R^{2C\times \frac{H}{4}\times \frac{W}{4}}$:

(11) $$F_{\mathrm{concat}}=\mathrm{Concat}\left(\left(F_{s,1}\right),\mathrm{Up}\left(F_{s,2}\right)\right),$$

where Concat(·) denotes the channel-wise concatenation operation, and Up(·) represents the upsampling operation.

The concatenated feature $F_{\mathrm{concat}}$ now contains redundant information and a doubled channel dimension. We use a lightweight 1×1 convolution, denoted as a fusion convolution ${\mathrm{Conv}}_{\mathrm{fuse}}$, to reduce the channel dimension back to *C* and adaptively merge the features from the two scales:

(12) $$F_{\mathrm{fuse}}={\mathrm{Conv}}_{\mathrm{fuse}}\left(F_{\mathrm{concat}}\right).$$

To refine the fused boundary features $F_{\mathrm{fuse}}$ and accentuate the most critical coastline information, we employ a Dual-Stream Attention Mechanism. This mechanism is designed to dynamically recalibrate the feature representation by concurrently processing it through two parallel streams: a Channel Attention Stream, which learns what features are important, and a Spatial Attention Stream, which identifies where the important features are located.

The Channel Attention Stream focuses on modeling the interdependencies between feature channels. The objective is to adaptively boost the feature channels that are most responsive to boundary-relevant patterns while suppressing less useful ones. This is achieved through a “Squeeze-and-Excitation” process. First, the “Squeeze” operation aggregates global spatial information into a channel descriptor using global average pooling. Then, the “Excitation” operation uses a lightweight Multi-Layer Perceptron (MLP) to learn a non-linear, channel-wise attention weight vector. The channel-refined feature map, $F_{c}$, is then produced by re-weighting the input features:

(13) $$F_{c}=F_{\mathrm{fuse}}⊙\sigma \left(\mathrm{MLP}\left({\mathrm{Pool}}_{\mathrm{avg}}\left(F_{\mathrm{fuse}}\right)\right)\right)$$

where ${\mathrm{Pool}}_{\mathrm{avg}}$ denotes the global average pooling operation, σ is the Sigmoid activation function, and ⊙ represents element-wise multiplication.

Simultaneously, the Spatial Attention Stream is responsible for identifying the most salient spatial regions. It generates a 2D spatial attention map to highlight pixels that are most likely part of the coastline and to diminish the importance of pixels in homogeneous inland or open-water areas. This is accomplished by applying a 1×1 convolutional layer to project the input features across the channel dimension, generating a spatial map that is then normalized by a Sigmoid function. The spatially refined feature map, $F_{s}$, is computed as:

(14) $$F_{s}=F_{\mathrm{fuse}}⊙\sigma \left({\mathrm{Conv}}_{1\times 1}\left(F_{\mathrm{fuse}}\right)\right)$$

where the convolution creates a weighted projection of features at each spatial location. Finally, to synthesize the complementary insights from both attention streams, we perform an adaptive fusion. The channel-refined features $F_{c}$ and the spatial-refined features $F_{s}$ are concatenated along the channel dimension. This combined feature map is then fed into a 1×1 convolutional layer, which learns the optimal way to merge the two streams, producing the final refined feature map $F_{\mathrm{refined}}$:

(15) $$F_{\mathrm{refined}}={\mathrm{Conv}}_{1\times 1}^{\mathrm{fuse}}\left(\mathrm{Concat}\left(\left[F_{c},F_{s}\right]\right)\right)$$

This dual-stream architecture ensures that the features passed to the final prediction layer are highly focused, benefiting from both channel-wise discrimination and precise spatial localization, which is crucial for accurate coastline delineation.

Subsequently, a 1×1 prediction convolution, ${\mathrm{Conv}}_{\mathrm{pred}}$, is applied to the refined feature map $F_{\mathrm{refined}}$ to generate the final coastline prediction map:

(16) $$E={\mathrm{Conv}}_{\mathrm{pred}}\left(F_{\mathrm{refined}}\right),$$

where $E\in R^{2\times \frac{H}{4}\times \frac{W}{4}}$ and the two output channels correspond to the logits for the boundary and non-boundary classes, respectively.

In summary, the Boundary Feature Fusion Unit effectively integrates multi-scale boundary information, reduces redundancy, and adaptively refines the resulting features through an attention mechanism. This ensures that the final coastline prediction is both detailed and robust, benefiting from the complementary characteristics of different feature levels.

### 3.3. Loss Function

The network proposed performs segmentation prediction by boundary head and segmentation head, respectively. For different task branches, we propose a segmentation loss function and a boundary loss function to jointly optimize the network parameters. In this part, the loss function used by the network is described in detail.

1. Segmentation loss function

In the segmentation head, the sea–land segmentation network can predict two categories: sea and land. For the sea–land segmentation binary classification task, the binary cross-entropy (BCE) loss function [48] is used to obtain the segmentation loss function, as shown in the following Equation (17):

(17) $$\begin{array}{c} L_{\mathrm{BCE}}=-\frac{1}{N}\sum_{i=1}^{N}\left(y_{i}\log\left({\hat{y}}_{i}\right)+\left(1-y_{i}\right)\log\left(1-{\hat{y}}_{i}\right)\right) \end{array}$$

where $L_{\mathrm{BCE}}$ represents the binary cross-entropy loss function, *y* and ${\hat{y}}_{i}$ represent the segmentation predicted result and label, *N* represents the total number of sample image pixels, and $y_{i}$ and ${\hat{y}}_{i}$ represent the segmentation predicted result and label of the *i*-th sample, respectively.

2. Boundary loss function

In the boundary head, the network can predict the boundary region and non-boundary in the image. However, the number of boundary pixels is much smaller than the number of non-boundary pixels, and there is the problem of class imbalance between boundary pixels and non-boundary pixels.

According to Equation (17), when the number of samples is unbalanced, the contribution of classes with a large number of samples to the total loss would be larger than that of classes with a small number of samples due to the lack of class weights. Specifically, the network can focus more on non-boundary regions when segmenting boundaries and ignore the boundary regions that need to be paid attention to. Therefore, the binary cross-entropy loss function cannot solve the problem of class imbalance in boundary segmentation. To solve this problem, a weighted binary cross-entropy loss function $L_{\mathrm{WBCE}}$ is introduced into the boundary loss function proposed in this algorithm. By adding a class weight to the binary cross-entropy loss function, it is helpful to improve the problem of class imbalance in boundary segmentation. The class loss weights were calculated as described in Algorithm 1.

In order to achieve effective constraints on the boundary regions, the proposed method adds the boundary-F1 loss function [25] to the boundary loss function to improve and optimize the quality of boundary segmentation. By calculating the boundary-F1 (BF1) score of the boundary prediction value and the boundary label value, 1-BF1 is used as the boundary-F1 loss function to achieve the purpose of optimizing the boundary.

The formulas for the $L_{\mathrm{WBCE}}$ and $L_{\mathrm{BF}1}$ loss functions are defined as Equations (18) and (19):

(18) $$\begin{array}{cc} L_{\mathrm{WBCE}}\left(y,\hat{y}\right) & =-\frac{1}{N}\sum_{i=1}^{N}(W_{1}\;y_{i}\log{\hat{y}}_{i}\\ \; & \;\;+W_{2}\;\left(1-y_{i}\right)\log\left(1-{\hat{y}}_{i}\right)). \end{array}$$

(19) $$\begin{array}{c} L_{BF1}=1-\frac{2\times P_{b}\times R_{b}}{P_{b}+R_{b}} \end{array}$$

where *N* represents the total number of pixels in the sample image, $y_{i}$ and ${\hat{y}}_{i}$ represent the segmentation prediction value and the true label value of the *i*-th sample, $W_{1}$ and $W_{2}$ represent the loss weight of two classes, respectively, and $P_{b}$ and $R_{b}$ represent the precision and recall rate of the predicted boundary pixel and the label within 2 pixels distance, respectively.
**Algorithm 1** Class loss weight calculation process.**Input:** *N* label images in the training set, number of classes *C* and specific categories *c* = 1,2,…,C **Output:** Class weight $W_{c}$ **Initialization:** 
1:The total number of label pixels was acquired (num = N×H×W), with a smoothing coefficient smooth = 1;2:**FOR i=1 : *N* DO**3:   Obtain the height *H* and the width *W* of the label image;4:   **FOR i=1 : *H* DO**5:      **FOR j=1 : *W* DO**6:         **IF** $label_{i,j}==c$7:            **THEN** calculates the total number of category pixels, $sum_{c}=sum_{c}+1$8:         **END IF**9:      **END FOR**10:   **END FOR**11:**END FOR**12:Computing the Class frequency: $freq_{c}=sum_{c}/sum$13:Obtain class weights: $W_{c}=1/log\left(freq_{c}+smooth\right)$14:Normalized class weights: $W_{c}=W_{c}/sum\left(W_{c}\right)$15:**RETURN** $W_{c}$


In summary, the boundary loss function proposed is defined as Equation (20):

(20) $$\begin{array}{c} L_{\mathrm{boundary}}=L_{\mathrm{WBCE}}\left(y,\hat{y}\right)+L_{\mathrm{BF}1}\left(y,\hat{y}\right) \end{array}$$

where $L_{\mathrm{boundary}}$ represents the boundary loss function, $L_{\mathrm{WBCE}}$ represents the weighted binary cross-entropy loss function, $L_{\mathrm{BF}1}$ represents the boundary-F1 loss function, and *y* and $\hat{y}$ represent the predicted result of boundary segmentation and the boundary labels, respectively.

3. Total loss function

Combining the loss functions described in the previous two subsections, the final total loss function $L_{total}$ of the network can be expressed as Equation (21).

(21) $$\begin{array}{c} L_{\mathrm{total}}=L_{\mathrm{seg}}+\lambda \cdot L_{\mathrm{boundary}} \end{array}$$

where λ is a hyperparameter used to balance the weights of the boundary loss function.

### 3.4. Implementation

We show the steps of the proposed method in Algorithm 2 to understand.
**Algorithm 2** Training Framework for Cross-Scale 3D Attention and Boundary Awareness Network.**Input:** Sea–Land port remote sensing image $I\in R^{H\times W\times C}$. **Output:** Predicted results of the validation set. **Initialization:**
1:Epoch set to 200; batch size set to 8; learning rate set to $3\times 10^{-4}$, and hyperparameter λ set to 0.7;2:Train the Cross-Scale 3D Attention Fusion Module and Boundary Awareness network for sea–land segmentation;3:**While i = 1 to n do:**4:Extract low, middle, and deep features from the ResNet-50 backbone;5:Input deep features to Cross-Scale 3D Attention Fusion Module to obtain an attention output $X_{attn}$;6:Input the shallow features to Boundary Awareness Enhancement Module to obtain feature $X_{b}$ then fuse $X_{attn}$ and $X_{b}$ to get $X_{fuse}$;7:Calculate segmentation loss and boundary loss between prediction results and ground truth;8:Validate the performance of the optimal weight on the validation set;9:Save the weights with the highest precision.10:**end for:**11:Obtain the saved weight and use the validation set with the trained model to achieve sea–land segmentation results.


## 4. Experiment

### 4.1. Dataset

In this section, the model is trained and evaluated on the following two public remote sensing image sea–land segmentation datasets:

1. Landsat-8 OLI dataset [49]: This dataset uses Landsat-8 OLI remote sensing images, covering about 18,000 square kilometers of coastline in coastal areas of China, including four different types of coastal areas, namely sandy coast, rocky coast, mudflat coast, and artificial coast. In our experiment, the optical images of this dataset are used, and a total of 3361 remote sensing sea and land images are collected. The resolution size is 512×512, including two categories of sea and land. In our experiment, the training set and the validation set of this sea–land segmentation dataset are randomly divided, and 2876 training set samples and 485 validation set samples are obtained.
2. SLSD dataset [18]: This dataset obtains 208 remote sensing sea and land images from Google Earth, with the size of about 1000×1000 pixels and the resolution between 3 and 5 m. In our experiment, the sea–land segmentation dataset is randomly cropped, rotated and flipped, and a total of 1163 training set samples and 164 validation set samples are obtained with a resolution of 512×512.

Note that each dataset provides a training split and a single held-out validation split, and no separate test split is defined. Following the common protocol of previous sea–land segmentation studies, the validation set is used both for checkpoint selection and for final evaluation, and this protocol is applied identically to all compared methods to ensure a fair comparison.

### 4.2. Evaluation Indicators

We take the following indicators as the main basis for evaluating the sea area segmentation effect: F1 score (F1), Overall Accuracy (OA), Mean Intersection over Union (mIoU), boundary-F1 score (BF), and Boundary Intersection over Union (BIoU). The BF score is computed with a pixel tolerance of 2, and the BIoU is calculated within a boundary band of the same width.

### 4.3. Experimental Environment

We conducted experiments on the land–sea segmentation model within the PyTorch framework on a local Linux server running Ubuntu. The hardware configuration comprised five NVIDIA Quadro RTX 3090 graphics cards (NVIDIA Corporation, Santa Clara, CA, USA), with experiments performed in a Python 3.8.13 environment.

### 4.4. Experiment Details

The experimental details of the proposed method are as follows: for the experimental parameters in the training stage on the two sea–land segmentation datasets, the total number of training iterations is set to 200 rounds, the training batch size is set to 8, the validation batch size is set to 2, and the weight of the boundary loss function is λ=0.7. ResNet50 in the backbone uses weight parameters pre-trained on the ImageNet [50] dataset. The network was trained by the Adam optimizer, the initial learning rate was set to $3\times 10^{-4}$, the learning rate adjustment strategy was Poly strategy, and the other modules were randomly initialized.

### 4.5. Experimental Results

In order to fully verify the effectiveness of the sea–land segmentation algorithm based on Cross-Scale 3D Attention and Boundary Awareness Enhancement Module proposed in our experiment, extensive experiments are carried out on Landsat-8 OLI and SLSD, two public remote sensing image sea–land segmentation datasets. In this part, the proposed sea–land segmentation model is compared with ten semantic segmentation models and three sea–land segmentation models, which are FCN [15], U-Net [14], PSPNet [51], Deeplabv3+ [52], DANet [53], HSTUNet [54], CRFNet [55], DBBANet [56], CMLFormer [28], and MCAT-UNet [20]. The three sea–land segmentation models are ACU-Net [57], DBENet [58], and HED-UNet [59]. The detailed comparative experimental results are as follows:

1. Comparative experimental results on Landsat-8 OLI dataset:

Table 1 shows the comparative experimental results of each model on the Landsat-8 OLI dataset, where the optimal results of each method are marked with bold font.

**Figure 5:**
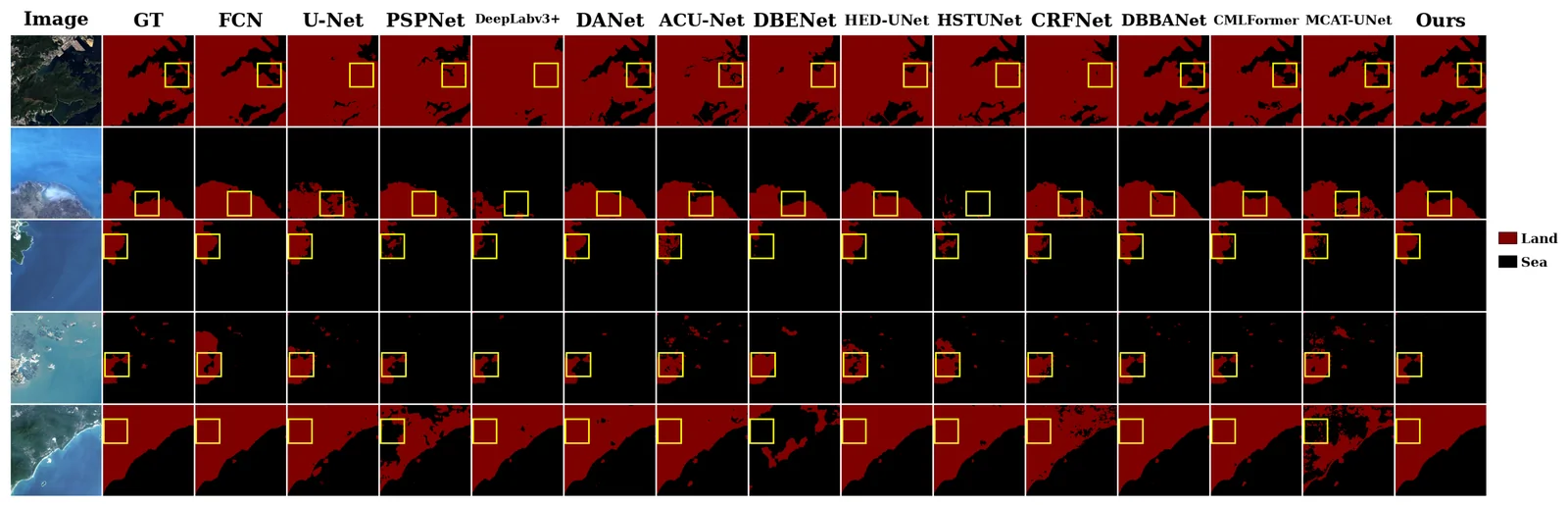
Visualization results of each comparison method on the Landsat-8 OLI dataset. The yellow boxes highlight the typical regions.

As shown in Table 1, the F1 score, OA, and mIoU of the proposed method reach 99.16%, 99.21%, and 98.33%, respectively, which are higher than those of all the comparison methods. Compared with DBBANet, the strongest baseline in terms of mIoU, the proposed method obtains improvements of 0.23% in mIoU and 0.11% and 0.12% in OA and F1, respectively. Compared with the two recently proposed Transformer-based baselines, the proposed method surpasses CMLFormer by 0.89% in mIoU and MCAT-UNet by 1.89%, with OA and F1 gains of 0.45%/0.94% and 0.46%/0.97%, respectively. More importantly, the advantage becomes much more pronounced on the boundary-specific metrics: the proposed method achieves a boundary-F1 of 28.29% and a boundary IoU of 67.07%, exceeding the strongest baseline DBBANet by 4.45 and 2.33 percentage points (i.e., relative improvements of 18.7% and 3.6%), and surpassing CMLFormer by 10.39 and 6.55 percentage points and MCAT-UNet by 11.75 and 16.04 percentage points, respectively. It is also worth noting that the general semantic segmentation models attain high region-level scores but very low boundary scores (e.g., Deeplabv3+ reaches 97.55% mIoU while its boundary-F1 is only 7.90%), which indicates that region-level metrics have become saturated on this dataset. Therefore, the substantial and consistent gains in boundary-F1 and boundary IoU provide direct quantitative evidence that the proposed boundary module genuinely improves coastline delineation rather than yielding only a marginal overall improvement. Figure 5 shows the visualization results of each comparison method on the Landsat-8 OLI dataset, where the proposed method yields more complete island and reef segmentation and smoother sea–land boundaries (yellow boxes), consistent with the quantitative results in Table 1.

2. Comparative experimental results on the SLSD dataset

Table 2 shows the comparative experimental results of each model on the SLSD dataset, where the optimal results of each method are marked in bold font. As shown in Table 2, the F1 score, OA, and mIoU of the proposed method reach 99.35%, 99.36%, and 98.72%, respectively, and the proposed method achieves the best results on all five evaluation metrics. Compared with DBBANet, the strongest baseline in terms of mIoU, the proposed method obtains improvements of 0.03% in mIoU and 0.11% in both OA and F1; compared with the two recently proposed Transformer-based baselines, it surpasses CMLFormer by 0.11% in mIoU and MCAT-UNet by 0.64%, with F1 and OA gains of 0.05%/0.06% and 0.32%/0.33%, respectively. On the boundary-specific metrics, the proposed method achieves a BF of 56.84% and a BIoU of 82.93%, exceeding DBBANet by 0.63 and 2.46 percentage points, CMLFormer by 4.98 and 2.55 percentage points, and MCAT-UNet by 8.60 and 9.37 percentage points, respectively. It is worth noting that the boundary scores of all methods on the SLSD dataset are substantially higher than those on the Landsat-8 OLI dataset (Table 1), since the higher spatial resolution of SLSD (3–5 m) renders the sea–land boundaries clearer; even under this more favorable condition, the proposed method still maintains the best performance on all metrics. Furthermore, compared with the existing sea–land segmentation methods, the proposed algorithm is 1.05% higher than DBENet in mIoU, with the OA and F1 scores increased by 0.54% and 0.53%, respectively.

**Figure 6:**
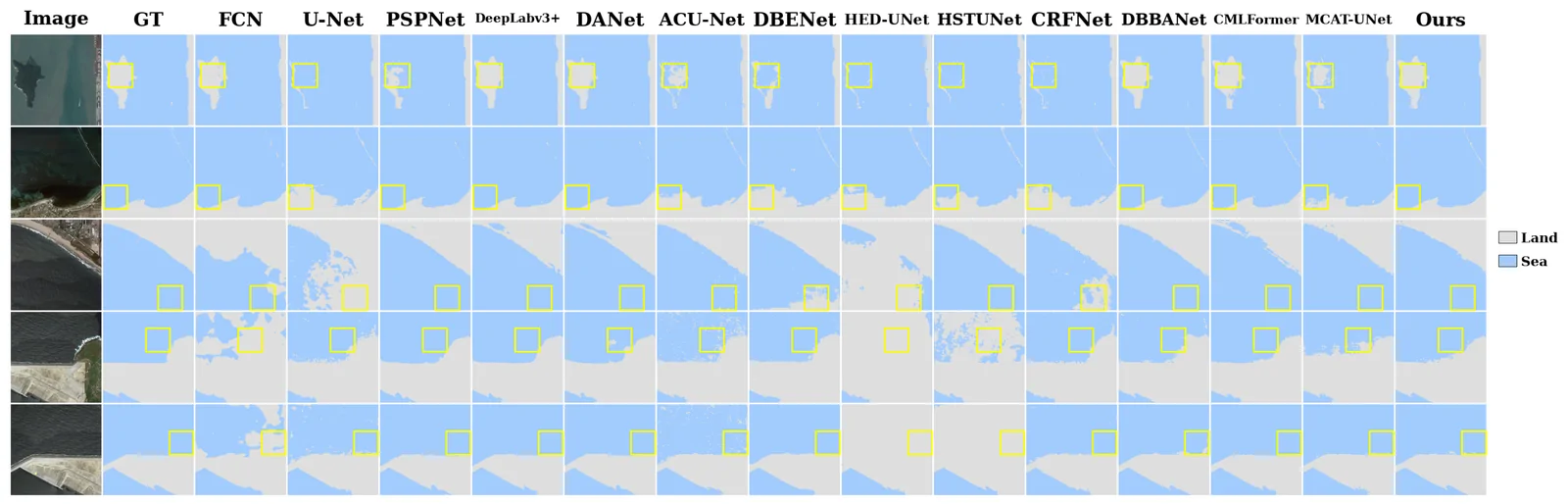
Visualization of each comparison method on the SLSD dataset.

Figure 6 shows the visualization results of each comparison method on the SLSD dataset. From the yellow box area in the first column, it can be observed that in the artificial coast type breakwater area, the DBBANet model has a poor segmentation effect in the segmentation breakwater area, and the breakwater has a discontinuity problem. The U-Net and HED-UNet can accurately segment the breakwater, but the fineness is not good when segmenting the targets in the breakwater area. The model in our experiment can segment the targets of breakwater and breakwater area at the same time, and the segmentation fineness is better than other comparison methods. From the yellow box area in the second column, it can be observed that the proposed method can accurately identify the inland river in the intersection area of the sea and the river, which ensures the connectivity between the sea and the land. It can be observed from the yellow box area of the third to the fifth column that in the wharf area of the artificial coast type, the U-Net, DBBANet, and HED-UNet misclassified part of the sea area in the wharf area into the land area, resulting in poor segmentation results. However, the boundary continuity and segmentation effect of the model in our experiment are more accurate in the sea–land wharf area. It is superior to other comparison methods.

Table 3 and Table 4 compare the computational cost of all methods on the Landsat-8 OLI and SLSD datasets, respectively, measured with an input size of 512 × 512 and a batch size of 1. Owing to the decomposed three-plane attention, the FLOPs of the proposed method (124.85 G) remain lower than those of several representative baselines (e.g., DANet, 209.75 G; PSPNet, 177.37 G; CRFNet, 160.30 G) despite the additional pseudo-3D attention and boundary branch. Its inference speed reaches 59.2 and 59.6 FPS on the two datasets, which satisfies the common 30 FPS real-time threshold and is comparable to DeepLabv3+ (64.0 FPS). The main cost lies in the number of parameters (55.09 M), which is higher than that of the baselines because the pseudo-3D volume and the boundary branch introduce additional convolutional layers; even so, the peak GPU memory (515/525 MB) remains lower than that of U-Net (577 MB) and ACU-Net (622/621 MB). Considering that the proposed method achieves the best results on all five metrics on both datasets, with the most notable gain being a 4.45-percentage-point improvement in boundary-F1 over DBBANet on the Landsat-8 OLI dataset, this computational overhead is an acceptable trade-off for the substantially improved boundary quality. Reducing the parameter count through lightweight design is left as future work.

### 4.6. Ablation Study

In order to verify the importance and effectiveness of each module in the sea–land segmentation algorithm proposed in our method, we primarily select the Landsat-8 OLI sea–land segmentation dataset as the experimental dataset to conduct ablation experiments. The ablation experiments are as follows:

1. Comparative results of different attention

In order to better reflect the superiority of our designed contextual attention for the feature recognition of sea–land scenes, we replaced it with Nonlocal [60], CBAM [61], SCSE [62], CoT [63], and SWSAM [64] respectively, and compared the results. As shown in Table 5, our Cross-Scale 3D Attention outperforms these alternatives in establishing long-range dependency and achieves the optimal performance on all three metrics.

2. Effectiveness of different modules of the network

In order to verify the effects of different modules in the proposed algorithm, we set up multiple sets of comparative experiments for verification, including the following experimental groups: (a) only using the backbone and decoder; (b) adding the Cross-Scale 3D Attention Module; (c) adding the Boundary Awareness Enhancement Module; (d) the Cross-Scale 3D Attention Module and the Boundary Awareness Enhancement Module are added at the same time.

As shown in Table 6, the Cross-Scale 3D Attention Module contributes the most to the sea–land segmentation results, and the introduction of the Cross-Scale 3D Attention Module can improve the mIoU from 96.25% to 98.08%. In the feature extraction process, the backbone primarily focuses on the details and local information of the image.

Adding the Boundary Awareness Enhancement Module can improve the mIoU from 96.25% to 98.06%. The proposed method also adds the Cross-Scale 3D Attention Module and the Boundary Awareness Enhancement Module, which can improve the mIoU to 98.33%.

In summary, it can be concluded that the Cross-Scale 3D Attention Module is more important for sea–land segmentation, and the global context features extracted by this module can improve the model’s attention to the sea–land coastal zone. The Boundary Awareness Enhancement Module can also improve the accuracy of the model, so that the model can improve the segmentation effect of the sea–land boundary region.

To further isolate the contribution of each unit inside the Boundary Awareness Enhancement Module, we incrementally add its three units to the configuration containing the backbone, the decoder, and the Cross-Scale 3D Attention Module (Baseline): the Boundary Awareness Unit (BAU, Sobel-guided boundary extraction), the Contextual Enhancement Unit (CEU, context-guided boundary refinement), and the Boundary Feature Fusion Unit (BFFU, multi-scale fusion with dual-stream attention). As shown in Table 7, BAU injects an explicit boundary prior and improves all metrics on both datasets at a negligible parameter cost (+0.70 M); CEU, which modulates the boundary features with the attention-enhanced deep semantics, contributes the largest BF gain on Landsat-8 OLI (+1.12 points, together with +1.59 points in BIoU) and a further +3.11 points on SLSD, confirming that semantic guidance is the key ingredient for accurate boundary localization; and BFFU fuses the multi-scale boundary features and brings the full model to the best scores on all six metrics on both datasets. The entire boundary module adds only 1.00 M parameters (54.09 M to 55.09 M).

3. Analysis on the Fusion of Features

When considering which specific features to fuse, multiple approaches exist. For instance, fusing all four features is possible, but the features of C1 and C4 differ significantly in semantic meaning. C1 focuses on pixel-level patterns, while C4 focuses on large-area categories. Forcibly merging them into a unified attention space may make learning difficult for the model or even produce negative effects (semantic noise interfering with details, or detail noise interfering with semantics). Another approach involves merging only two features. Merging C2 and C4 while skipping C3 results in an insufficiently smooth information flow, forcing the model to work harder to bridge the large semantic gap between C2 and C4. Merging C3 and C4 is a viable solution, as it requires less computation and memory than merging all three layers, while C3 and C4 are the two layers richest in semantic information. Merging these layers enhances the model’s understanding of large-scale regional attributes, aiding in distinguishing complex scenes (e.g., murky coastal waters and wetlands). However, the loss of mid-level information may result in reduced segmentation performance for medium-sized islands or intricate harbors.

Table 8 presents the experimental details of integrating different features. The results indicate that incorporating all four features yields lower performance than the other two approaches. Furthermore, adding the intermediate-level feature C2 produces slightly better results than integrating only C3 and C4.

4. Comparison of different boundary loss functions

In order to verify the effectiveness of the boundary loss function in the proposed algorithm, we combine different loss functions as the boundary loss function, and verify the feasibility of the boundary loss function proposed in our method through multiple sets of comparative experiments. The loss functions used in the experiments are as follows: unweighted binary cross-entropy loss function $L_{\mathrm{BCE}}$, weighted binary cross-entropy loss function $L_{\mathrm{WBCE}}$, boundary-F1 loss function $L_{\mathrm{BF}1}$, Dice loss function $L_{\mathrm{Dice}}$, and IoU loss function $L_{\mathrm{IoU}}$.

Table 9 shows the experimental results of different combinations of boundary loss functions on the Landsat-8 OLI dataset. The weighted binary cross-entropy loss function $L_{\mathrm{WBCE}}$ proposed in our method has a 0.2% improvement in the mIoU evaluation index compared with the ordinary binary cross-entropy loss function $L_{\mathrm{BCE}}$; adding the boundary-F1 loss further raises the mIoU to 97.83% ($L_{\mathrm{BCE}}+L_{\mathrm{BF}1}$) and 98.33% ($L_{\mathrm{WBCE}}+L_{\mathrm{BF}1}$, the best), while $L_{\mathrm{WBCE}}+L_{\mathrm{IoU}}$ yields a smaller gain (97.75%), indicating a weaker constraint on thin boundary structures. The sharp drop of $L_{\mathrm{WBCE}}+L_{\mathrm{Dice}}$ (F1 = 92.85%) stems from the instability of the Dice term on extremely imbalanced boundary maps (boundary pixels account for less than 2% of all pixels): its gradients oscillate severely in the early epochs, and the model converges to an under-fitted solution. These results justify the proposed $L_{\mathrm{WBCE}}+L_{\mathrm{BF}1}$ combination.

Although the performance improvement may not be significant on some metrics, the proposed network excels in handling challenging cases, such as complex backgrounds and weak boundaries, where other models tend to struggle. This robustness is crucial for real-world applications in sea–land segmentation.

## 5. Conclusions

This paper proposes CABANet, a sea–land segmentation network that combines a Cross-Scale 3D Attention Fusion Module with a Boundary Awareness Enhancement Module for coastal areas with complex terrain and weak boundary continuity. The former stacks multi-scale features into a pseudo-3D volume and models cross-scale dependencies through decomposed attention on three orthogonal planes, at a computational cost that grows linearly with the volume size; the latter enhances boundary features through Sobel-guided extraction, context-guided refinement, and multi-scale fusion, and is optimized jointly with the segmentation task via a weighted cross-entropy and boundary-F1 loss. Experiments on two public sea–land segmentation benchmarks show that the proposed method achieves the best results on all metrics, with ablation studies verifying the contribution of each component.

Nevertheless, several limitations should be acknowledged: the margin over the strongest baseline on SLSD is small (0.03% in mIoU; Table 2); the two modules are coupled, as shown by the interaction effect in Table 6; and the network introduces additional parameters (55.09 M; Table 3). Future work will therefore focus on lightweight design to reduce this overhead, multi-seed stability analysis and geographically independent evaluation to solidify the reliability and generalization of the reported improvements, and validation in more diverse coastal environments.

## Figures and Tables

**Table 1:** Comparative experimental results of each model on Landsat-8 OLI dataset. The best results are shown in bold font.

Dataset	Methods	F1/%	OA/%	mIoU/%	BF/%	BIoU/%
Landsat-8 OLI	FCN [15]	98.35	98.43	96.76	12.15	52.88
	U-Net [14]	98.32	98.40	96.70	20.58	50.57
	PSPNet [51]	98.67	98.74	97.38	12.86	50.54
	Deeplabv3+ [52]	98.76	98.82	97.55	7.90	47.33
	DANet [53]	98.65	98.72	97.34	13.11	53.47
	ACU-Net [57]	98.30	98.38	96.66	17.76	50.61
	DBENet [58]	98.50	98.57	97.03	9.63	34.41
	HED-UNet [59]	98.50	98.57	97.04	20.32	57.37
	CRFNet [55]	98.31	98.41	97.10	17.25	51.19
	HSTUNet [54]	98.38	98.45	97.81	13.47	49.40
	DBBANet [56]	99.04	99.10	98.10	23.84	64.74
	CMLFormer [28]	98.70	98.76	97.44	17.90	60.52
	MCAT-UNet [20]	98.19	98.27	96.44	16.54	51.03
	Proposed	**99.16**	**99.21**	**98.33**	**28.29**	**67.07**

**Table 2:** Comparative experimental results of each model on SLSD dataset. The best results are shown in bold font.

Dataset	Methods	F1/%	OA/%	mIoU/%	BF/%	BIoU/%
SLSD	FCN [15]	97.41	97.40	94.94	20.58	62.21
	U-Net [14]	98.84	98.84	97.71	40.02	70.65
	PSPNet [51]	98.99	99.11	98.05	32.22	73.50
	Deeplabv3+ [52]	99.03	99.04	98.10	38.68	77.64
	DANet [53]	98.96	98.97	97.96	35.45	74.15
	ACU-Net [57]	98.72	98.72	97.47	37.64	64.93
	DBENet [58]	98.82	98.82	97.67	47.92	70.93
	HED-UNet [59]	97.37	97.32	94.80	47.27	68.38
	CRFNet [55]	98.68	98.68	97.40	44.64	71.64
	HSTUNet [54]	98.60	98.61	97.25	23.78	71.63
	DBBANet [56]	99.24	99.25	98.69	56.21	80.47
	CMLFormer [28]	99.30	99.30	98.61	51.86	80.38
	MCAT-UNet [20]	99.03	99.03	98.08	48.24	73.56
	Proposed	**99.35**	**99.36**	**98.72**	**56.84**	**82.93**

**Table 3:** Computational cost comparison of different methods on the Landsat-8 OLI dataset. All models are tested with an input size of 512 × 512 and a batch size of 1. The best results are shown in bold font.

Methods	Params (M)	FLOPs (G)	FPS	Memory (MB)
FCN [15]	15.31	80.33	129.6	254
U-Net [14]	13.39	124.05	69.0	577
PSPNet [51]	48.63	177.37	59.7	469
Deeplabv3+ [52]	41.68	163.85	64.0	308
DANet [53]	49.48	209.75	50.3	395
ACU-Net [57]	22.31	132.91	64.8	622
DBENet [58]	18.34	93.24	70.9	485
HED-UNet [59]	**8.13**	14.67	**244.5**	**172**
CRFNet [55]	17.27	160.30	58.4	615
HSTUNet [54]	31.36	50.11	35.2	294
DBBANet [56]	36.01	62.77	101.9	349
CMLFormer [28]	12.53	**14.29**	130.9	174
MCAT-UNet [20]	22.17	18.41	86.0	204
Proposed	55.09	124.85	59.2	515

**Table 4:** Computational cost comparison of different methods on the SLSD dataset. All models are tested with an input size of 512 × 512 and a batch size of 1. The best results are shown in bold font.

Methods	Params (M)	FLOPs (G)	FPS	Memory (MB)
FCN [15]	15.31	80.33	129.6	254
U-Net [14]	13.39	124.05	69.0	577
PSPNet [51]	48.63	177.37	59.7	469
Deeplabv3+ [52]	41.68	163.85	64.0	308
DANet [53]	49.48	209.75	50.3	395
ACU-Net [57]	22.31	132.91	64.7	621
DBENet [58]	18.34	93.24	70.6	483
HED-UNet [59]	**8.13**	14.67	**243.6**	**172**
CRFNet [55]	17.27	160.30	58.4	615
HSTUNet [54]	31.36	50.11	35.2	294
DBBANet [56]	36.01	62.77	101.9	356
CMLFormer [28]	12.53	**14.29**	127.7	174
MCAT-UNet [20]	22.17	18.41	82.1	204
Proposed	55.09	124.85	59.6	525

**Table 5:** Experimental results of different attention mechanisms on the Landsat-8 dataset. The best results are shown in bold font.

Dataset	Attention	F1/%	OA/%	mIoU/%
Landsat-8 OLI	NonLocal [60]	98.48	98.56	97.01
	CBAM [61]	98.55	98.62	97.14
	SCSE [62]	98.74	98.80	97.51
	CoT [63]	98.47	98.55	97.00
	SWSAM [64]	98.63	98.70	97.31
	Proposed	**99.16**	**99.21**	**98.33**

**Table 6:** Ablation experimental results of each network module on the Landsat-8 OLI dataset. A check mark (✓) indicates that the corresponding module is adopted in the model. The best results are shown in bold font.

Dataset	Method Description		F1/%	OA/%	mIoU/%
	Cross-Scale 3D Attention	Boundary Awareness Enhancement			
Landsat-8 OLI			98.06	98.18	96.25
	✓		99.03	99.09	98.08
		✓	99.02	99.08	98.06
	✓	✓	**99.16**	**99.21**	**98.33**

**Table 7:** Ablation results of the individual components of the Boundary Awareness Enhancement Module on the Landsat-8 OLI and SLSD datasets. The best results are shown in bold font.

Configuration	Landsat-8 OLI			SLSD			Params (M)
	mIoU/%	BF/%	BIoU/%	mIoU/%	BF/%	BIoU/%	
Baseline	98.08	26.50	64.58	98.55	49.85	80.28	54.09
+ BAU	98.26	26.98	65.19	98.59	53.68	81.57	54.79
+ BAU + CEU	98.26	28.10	66.78	98.69	56.79	81.94	55.06
+ BAU + CEU + BFFU (full)	**98.33**	**28.29**	**67.07**	**98.72**	**56.84**	**82.93**	**55.09**

**Table 8:** Experimental results of different feature combinations on the Landsat-8 OLI dataset. The best results are shown in bold font.

Dataset	Method	F1/%	OA/%	mIoU/%
Landsat-8 OLI	$C_{3}+C_{4}$	99.14	99.19	98.29
	$C_{1}+C_{2}+C_{3}+C_{4}$	99.08	99.14	98.19
	$C_{2}+C_{3}+C_{4}$	**99.16**	**99.21**	**98.33**

**Table 9:** Experimental results of different boundary loss functions on the Landsat-8 OLI dataset. The best results are shown in bold font.

Dataset	Method	F1/%	OA/%	mIoU/%
Landsat-8 OLI	$L_{\mathrm{BCE}}$	98.51	98.57	97.09
	$L_{\mathrm{WBCE}}$	98.71	98.64	97.33
	$L_{\mathrm{BCE}}+L_{\mathrm{BF}1}$	99.03	99.10	97.83
	$L_{\mathrm{WBCE}}+L_{\mathrm{Dice}}$	92.85	93.19	92.42
	$L_{\mathrm{WBCE}}+L_{\mathrm{IoU}}$	99.06	98.98	97.75
	$L_{\mathrm{WBCE}}+L_{\mathrm{BF}1}$	**99.16**	**99.21**	**98.33**

## Data Availability

The Landsat-8 OLI dataset and the SLSD dataset used in this study are publicly available. The corresponding references are cited in Section 4.1. The original data and code of CABANet are openly available in the GitHub repository at https://github.com/Lizzie-yan/CABANet (version 1.0, accessed on 15 October 2026).
